# Supplementary material for: Development of a welfare assessment tool for tourist camp elephants in Asia
Source: PeerJ. 2024 Oct 28;12:e18370. doi: 10.7717/peerj.18370 (PMC11526799; doi:10.7717/peerj.18370)
Supplement: Supplemental Information 3 [file peerj-12-18370-s003.docx]

**VALIDATION OF ELEPHANT WELFARE ASSESSMENT TOOL: A Content Validity Test**

**Dear experts,**

We are planning to develop a welfare assessment tool for tourist camp elephants using the Five Domains model (Mellor et al., 2020), incorporating animal-based and resource-based measures, and utilizing non-invasive methods such as direct observation and interviews. The Five Domains model will enable a more nuanced and accurate assessment of their overall well-being by considering multiple domains, including nutrition, environment, health, behavioral interactions, and the animal's subjective experiences (mental state).

For that, we have developed a welfare assessment form which will be used to collect the individual data of captive elephants present in different elephant camps of Chiang Mai. This assessment form contains 5 domains and 39 measures related to the overall welfare of captive elephants across nutrition, environment, health, and joint approach of behavior and mental state domain. We need your expert judgment on the degree of relevance of each item to the measured domains. Please be as objective and constructive as possible in your review and use the following rating scale:

**Degree of relevance**

1 = The item is not relevant to the measured domain

2 = The item is somewhat relevant to the measured domain

3 = The item is quite relevant to the measured domain

4 = The item is highly relevant to the measured domain

The major objective of this is to check your agreement/disagreement with our measure’s selection. You can agree and disagree with the measures we have selected. However, we request you be unbiased regarding the welfare measures and select the degree of relevance based on scientific knowledge and the relevance of elephants kept in “tourist venues”. This process is to provide evidence of content validity for our welfare assessment tool by computing a content validity index (CVI), based on experts’ ratings of item relevance.

**TOOL VALIDATION FORM**

Please circle the degree of relevance of each item to the measured domains.

| No. | Measures | Degree of relevance | | | | Comments / Suggestions |
| --- | --- | --- | --- | --- | --- | --- |
|  | **NUTRITION** |  | | | |  |
| 1. | Feed Variety | 1 | 2 | 3 | 4 |  |
| 2. | Feed Frequency | 1 | 2 | 3 | 4 |  |
| 3. | Feed Freshness | 1 | 2 | 3 | 4 |  |
| 4. | Water Frequency | 1 | 2 | 3 | 4 |  |
| 5. | Water Quality | 1 | 2 | 3 | 4 |  |
|  | **ENVIRONMENT** |  | | | |  |
| 6. | Chain Length / Enclosure Space (Day & Night) | 1 | 2 | 3 | 4 |  |
| 7. | Shade (Day & Night) | 1 | 2 | 3 | 4 |  |
| 8. | Hygiene (Day & Night) | 1 | 2 | 3 | 4 |  |
| 9. | Noise Type (Day & Night) | 1 | 2 | 3 | 4 |  |
| 10. | Substrate (Day & Night) | 1 | 2 | 3 | 4 |  |
| 11. | Access to Social Interaction | 1 | 2 | 3 | 4 |  |
| 12. | Environment Complexity /Enrichment | 1 | 2 | 3 | 4 |  |
| 13. | Access to Bathing | 1 | 2 | 3 | 4 |  |
| 14. | Restriction Time | 1 | 2 | 3 | 4 |  |
| 15. | Access to Foraging | 1 | 2 | 3 | 4 |  |
| 16. | Opportunity to Mate | 1 | 2 | 3 | 4 |  |
| 17. | Duration of Mahout-Elephant Working Relationship | 1 | 2 | 3 | 4 |  |
| 18. | Mahout Job Satisfaction / Mahout Welfare (0 being the worst and 10 being the best) | 1 | 2 | 3 | 4 |  |
| 19. | Use of Ankus | 1 | 2 | 3 | 4 |  |
|  | **HEALTH** |  | | | |  |
| 20. | Body Condition Score (BCS) | 1 | 2 | 3 | 4 |  |
| 21. | Nail Score | 1 | 2 | 3 | 4 |  |
| 22. | Wound Score | 1 | 2 | 3 | 4 |  |
| 23. | Eye Condition | 1 | 2 | 3 | 4 |  |
| 24. | Skin Condition | 1 | 2 | 3 | 4 |  |
| 25. | Health Care | 1 | 2 | 3 | 4 |  |
| 26. | Exercise Hours | 1 | 2 | 3 | 4 |  |
| 27. | Locomotion / Walking Pattern | 1 | 2 | 3 | 4 |  |
| 28. | Urine and Feces Condition | 1 | 2 | 3 | 4 |  |
| 29. | Mucous Membrane Condition | 1 | 2 | 3 | 4 |  |
|  | **BEHAVIOR AND MENTAL STATE** |  |  |  |  |  |
| 30. | Foraging / Feeding Behavior | 1 | 2 | 3 | 4 |  |
| 31. | Rest/Sleep Behavior | 1 | 2 | 3 | 4 |  |
| 32. | Conspecific Interaction | 1 | 2 | 3 | 4 |  |
| 33. | Human-Elephant Interaction (Mahout) | 1 | 2 | 3 | 4 |  |
| 34. | Human-Elephant Interaction (Tourist) | 1 | 2 | 3 | 4 |  |
| 35. | Elephant General State (Restricted State) | 1 | 2 | 3 | 4 |  |
| 36. | Stereotypies (Restricted State) | 1 | 2 | 3 | 4 |  |
| 37. | Elephant General State (Unrestricted State) | 1 | 2 | 3 | 4 |  |
| 38. | Comfort or Self-Maintenance Behavior (Unrestricted State)   - Rubbing with a tool - Scratching the body on surfaces - Throwing straw in the body - Body slap with trunk | 1 | 2 | 3 | 4 |  |
| 39. | Comfort or Self-Maintenance Behavior (Unrestricted State)   - Water bath - Dust bath - Rolling in mud | 1 | 2 | 3 | 4 |  |
