# Supplementary material for: Development of a welfare assessment tool for tourist camp elephants in Asia
Source: PeerJ. 2024 Oct 28;12:e18370. doi: 10.7717/peerj.18370 (PMC11526799; doi:10.7717/peerj.18370)
Supplement: Supplemental Information 4 [file peerj-12-18370-s004.docx]

| **Grading System** | **Animal-Based Measures** | **Resource-Based Measures** |
| --- | --- | --- |
| Score 0 | Welfare Concern (Not Adequate) | High-Risk |
| Score 1 | Potential Welfare Concern (Needs Improvement) | Moderate-Risk |
| Score 2 | No Welfare Concern (Adequate) | Low-Risk |

**Elephant Welfare Assessment Tool - Sampling Form**

**Phase I: Interview with Mahouts**

| Date |  |
| --- | --- |
| Elephant Camp Name |  |
| Elephant Name |  |
| Mahout Name |  |
| Age |  |
| Sex |  |
| Origin (Same or Different Facility) |  |
| Elephant Daily Tourist Interaction / Day |  |
| Camp Average Tourist Visit / Day |  |
| Tourist Activities Involved |  |

1. **How many varieties of harvested roughage/grass are provided daily? (Feed Variety)**

Score 0: 1-2

Score 1: 3-4

Score 2: > 4

1. **How many times a day is an elephant provided food (Feed Frequency/Availability)?**

Score 0: 1-2 times in a fixed routine

Score 1: 3 or more times in a fixed routine

Score 2: Provided throughout the day in an unpredictable routine

1. **How fresh is the harvested roughage/grass (Feed Freshness)?**

Score 0: Pale and dry

Score 1: Slightly pale and partially green color, slightly moist

Score 2: Fresh green color and moist roughage

1. **How many times per day is water provided (Water Frequency)?**

Score 0: 1-2 times per day at fixed intervals

Score 1: 3 or more times at fixed intervals

Score 2: Water available ad libitum

1. **What is the condition of water provided (Water Quality)?**

Score 0: Turbid, stagnant, or contains dense foreign material (e.g., rotting leaves, plastics, elephant dung)

Score 1: Clear, but contains foreign material

Score 2: Clear, no foreign material

1. **What is the bathing interval (Access to Bathing)?**

Score 0: No daily baths/showers

Score 1: Daily bathing by the mahout from a pipe or hose

Score 2: Daily bathing in the river by free access or by the mahout

1. **How long is the elephant chained or placed in an enclosure space in the camp (Restriction Time/Movement/Choice and Control)?**

Score 0: > 12 hours/day

Score 1: 6-12 hours/ day

Score 2: < 6 hours/ day

1. **Does the elephant have access to forage in a nearby forest (Access to Foraging)?**

Score 0: No daily access

Score 1: Once a day

Score 2: Free access or multiple times a day

1. **What is the ankus used for? (Use of Ankus)**

Score 0: Regularly, to establish dominance and cause fear/distress (unjustified punishment)

Score 1: Regularly, direct elephant actions or if a verbal command is not effective

Score 2: Carried but only used in emergencies if elephant actions threaten mahout or tourist safety

1. **What kind of veterinary service is provided for the elephant (Health Care)?**

Score 0: No veterinary staff present or available locally

Score 1: Veterinary assistant/nurse is present or a veterinarian is on call if needed

Score 2: Trained veterinarian onsite

1. **How long does the elephant walk every day (including working time or other purposes) (Exercise Hours)?**

Score 0: < 1 hour/day

Score 1: 1-2 hours/day

Score 2: > 2 hours/day or free choice of movement

1. **Does the elephant lie down and sleep at night to rest (Rest/Sleep Behavior)?**

Score 0: Never lie down to sleep

Score 1: Sometimes lies down, but not every day

Score 2: Lies down to sleep every day

1. **How does the elephant interact with another elephant (Conspecific Interaction Behavior)?**

Score 0: Is aggressive to or fearful of other elephants

Score 1: Shows little interest or avoids interacting with other elephants

Score 2: Interested, playful, and relaxed with other elephants

1. **How does the elephant interact with the mahout (Human-Elephant Interaction)?**

Score 0: Aggressive to or fearful of its mahout

Score 1: Shows little interest or avoids interactions

Score 2: Playful, relaxed, interested in being around its mahout

1. **How does the elephant interact with tourists (Human-Elephant Interaction)?**

Score 0: Aggressive to or fearful of tourists

Score 1: Shows little interest or avoids interactions

Score 2: Willing to interact with tourists or no direct tourist interactions are provided

1. **Does the elephant show normal feeding behavior (Feeding Behavior)?**

Score 0: Shows little interest in food provided or foraging opportunities

Score 1: Consumes food provided by mahout

Score 2: Consumes food provided by mahout and forages independently

1. **What kind of enrichment is provided (Environment Complexity/Enrichment)?**

Score 0: No enrichment -provided

Score 1: At least 1 enrichment item (e.g., tree, pole, mud pool, water source) in the environment or mahout-provided (objects to interact with)

Score 2: >1 enrichment, either in the environment or mahout-provided

**Phase II: Observation of management practices**

**Daytime**

1. **How long is the chaining duration and in what area is the elephant chained? (Chain Length/Enclosure Space)**

Score 0: 0-5 m chain length or enclosure space of < 80 m^2^ (9 × 9m)

Score 1: 5-10 m chain length or enclosure space of 80 - 315 m^2^ (9 × 9 to 18 × 18m)

Score 2: More than 10 m chain length or enclosure space of more than 315 m^2^ (18 × 18m)

1. **How do elephants interact in the rest area (Access to Social Interaction)?**

Score 0: No direct contact

Score 1: Physical contact (e.g., trunk, body) with at least one elephant

Score 2: Can freely interact with one or more elephants

1. **What is the surface of the rest area? (Substrate)**

Score 0: Concrete

Score 1: Dirt, grass, or sand

Score 2: Choice of multiple substrates (Dirt, grass, or sand)

1. **Are there old feces/bad smells in the daytime area? (Hygiene)**

Score 0: Dirty area, bad smell, presence of old feces within one body length of the elephant

Score 1: No smell, but feces are stored within 1-5 body lengths of the elephant

Score 2: No smell, feces are removed regularly and stored more than 5 body lengths away from the elephant

1. **What kind of noise is around the day resting area? (Noise Type)**

Score 0: Large crowds, direct exposure to traffic, or other noise

Score 1: Occasional crowd noise, little electronic or traffic noise

Score 2: Only natural sounds

1. **What kind of shade does the animal have access to (Shade)?**

Score 0: No shade or covering

Score 1: Partial covering (e.g., single tree, mesh, net roof, etc.)

Score 2: Complete covering (e.g., naturally dense canopy, fixed, solid material roof)

**Nighttime**

1. **How long is the chaining duration, and in what area is the elephant chained? (Chain Length/Enclosure Space/Confinement)**

Score 0: 0-5 m chain length or enclosure space of < 80 m^2^ (9 × 9m)

Score 1: 5-10 m chain length or enclosure space of 80 - 315 m^2^ (9 × 9 to 18 × 18m)

Score 2: More than 10 m chain length or enclosure space of more than 315 m^2^ (18 × 18m)

1. **How do elephants interact at night (Access to Social Interaction)?**

Score 0: No direct contact

Score 1: Physical contact (e.g., trunk, body) with at least one elephant

Score 2: Can freely interact with one or more elephants

1. **What is the surface of the elephant nighttime area? (Substrate)**

Score 0: Concrete

Score 1: Dirt, grass, or sand

Score 2: Choice of multiple substrates (Dirt, grass, or sand)

1. **Are there old feces/bad smells in the nighttime area? (Hygiene)**

Score 0: Dirty area, bad smell, presence of old feces within one body length of the elephant

Score 1: No smell, but old feces are within 1-5 body length of the elephant

Score 2: Clean area, no smell, old feces are more than 5 body lengths away from the elephant

1. **What is the noise level? (Noise Type)**

Score 0: Direct exposure to traffic or other noise

Score 1: Occasional traffic noise

Score 2: Only natural sounds

1. **What kind of protection (Shade)?**

Score 0: No shade or covering

Score 1: Partial covering (e.g., single tree, mesh, net roof, etc.)

Score 2: Complete covering (e.g., naturally dense canopy, fixed, solid material roof)

**Phase III: Physical Examination**

1. **What is the body condition of the animal (Body Condition Score)?**

1 = thin; 5 = fat

Score 0: BCS = 1,5

Score 1: BCS = 2,4

Score 2: BCS = 3

1. **What is the condition of the nails (Nail Score)?**

Score 0: Complicated cracks (nail cracks exposing underlying tissue), overgrowth of nails or cuticles, dry cuticles, infection, moderate or severe injuries, nail loss

Score 1: Uncomplicated cracks (small cracks that do not extend into the cuticle) mild overgrowth of nails or cuticles, mild dry cuticles, mild disfigured nails, or mild injuries

Score 2: No lesions, normal nails

1. **Is there any wound present in the elephant’s body (Wound Score)?**

Score 0: Major wounds such as bleeding, infection with pus, deep destruction of tissue, exposed muscle or bone

Score 1: Minor wounds such as scrapes or scratches, no discharge

Score 2: No lesions

1. **What is the condition of skin (Skin Condition)?**

Score 0: Fungal infections, hyperkeratosis, rash, warts/ectoparasites

Score 1: Cracked, or peeling skin, mild hyperkeratosis

Score 2: Firm and wrinkled skin

1. **What is the condition of the eye (Eye Condition)?**

Score 0: Severe condition including discharge, ulcer, cataracts, opaqueness, swelling

Score 1: Mild tearing or redness

Score 2: Clear and bright eye, no discharge

1. **How well does the elephant walk? (Locomotion/Walking Pattern)?**

Score 0: Reluctant to move, exhibits evidence of severe pain while walking

Score 1: Mild lameness

Score 2: Normal gait

1. **What is the condition of feces and urine (Urine and Feces Condition)?**

Score 0: Bloody feces, diarrhea, constipation, parasites; bloody or turbid urine

Score 1: Coarse, dry feces; slightly dark yellow urine, no blood or pus

Score 2: Normal-shaped, moist fecal bolus; colorless to straw color urine, no blood or pus

1. **What is the condition of the mucous membrane at the trunk tip (Mucous Membrane Condition)?**

Score 0: Pale or white, dry mucous membrane at the tip of the trunk

Score 1: Pale pink, slightly dry mucous membrane at the tip of the trunk

Score 2: Bright pink, moist mucous membrane at the tip of the trunk

**Phase IV: Observation in the Chained State**

1. **What behaviors does the elephant exhibit during chaining (Elephant General State)?**

Score 0: Chain pulling, violent behavior towards people or other elephants that may include kicking, hitting with trunk, head pushing, charges, head shakes, distress vocalizations

Score 1: Tense body, head, ear, tail, or trunk; uninterested in surroundings and external stimuli

Score 2: Relaxed body, head, ear, and tail; regular use of trunk to investigate surroundings and respond positively to external stimuli

1. **Does the elephant exhibit stereotypic behavior during chaining (Stereotypic Behavior)?**

Score 0: Multiple times a day

Score 1: At least once a day

Score 2: None

**Phase V: Observation in the Non–Chained State**

1. **What is the behavior of the elephant when not chained (General Elephant State)?**

Score 0: Tense body, head, ear, tail, or trunk

Score 1: Relaxed, alert, responsive, movement of trunk, ear, and tail

Score 2: Curious and investigates the environment using trunk

1. **Comfort or Self-Maintenance Behavior (scratching body with a tool or on surfaces, throwing straw on back, and body slap with trunk): Among these behaviors, what does the elephant show currently?**

Score 0: None

Score 1: 1

Score 2: > 1

1. **Comfort or Self-Maintenance Behavior (water, dust bath and rolling in mud): Among these behaviors, what does the elephant show currently?**

Score 0: None

Score 1: 1

Score 2: > 1
